# Pain in women: bridging the gender pain gap

**DOI:** 10.1097/PR9.0000000000001276

**Published:** 2025-05-20

**Authors:** William Laughey, Katy Vincent, Samyuktha Iyer, Maria M. Cobo, Rebeccah Slater

**Affiliations:** a Health Professions Education Unit, Hull York Medical School, University of York, York, United Kingdom; b Reckitt Benckiser Healthcare Ltd, Dansom Lane South, Kingston Upon Hull, United Kingdom; c Nuffield Department of Women's and Reproductive Health, University of Oxford, Oxford, United Kingdom; d Department of Paediatrics, University of Oxford, Oxford, United Kingdom; e Colegio de Ciencias Biologicas y Ambientales, Universidad San Francisco de Quito USFQ, Quito, Ecuador

## Abstract

Bridging the gender pain gap requires collaborative efforts that address female-specific biological and psychosocial dimensions of pain through evidence-based, compassionate and empathy-driven approaches.

## 1. Introduction

Pain is more common in women than men. This is true for chronic pain^30^ and also for “everyday” pains, such as headaches and muscular pains.^17,32^ Research shows women experience an average of 1.6 painful events per month, compared with 1.2 for men (excluding sex-specific pains, such as dysmenorrhoea).^32^ For many clinical conditions that are associated with pain experiences, the female preponderance is well-established. Migraine, tension headache, fibromyalgia, irritable bowel syndrome, and autoimmune pain conditions such as rheumatoid arthritis are more common in women.^4^ Women are also generally more sensitive to pain from experimental noxious stimuli such as heat, cold and pressure.^17^ Despite women's higher prevalence of pain, evidence suggests it is taken less seriously, is subject to greater diagnostic delay, and is more likely to be undertreated than pain in men^3,6,9,15^—constituting a gender pain gap. In this perspective, we consider the biological and psychosocial dimensions of this gap, informed by the literature and insights gathered during multidisciplinary meetings with expertise spanning psychology, paediatrics, women's health, neurology and physiotherapy, and pharmacy. We highlight practical positive solutions, including unmasking the “hidden curriculum,” applying empathy and compassion scholarship to pain diagnosis and management, and building partnerships between academia, industry and government to drive progress.

## 2. Female-specific pains

In addition to the heightened prevalence and sensitivity of pain in women, there are also multiple female-specific pains, including dysmenorrhoea, endometriosis and fibroids, as well as pain-related to pregnancy, birth, and menopause. These pains are compounded by societal stigma and secrecy,^26^ and complicated by myth and misinformation, limiting open conversation, especially between sexes. These barriers to communication, together with misinformation among peer groups and on social media, make positive psychological framing of female pains more difficult and impair the adoption of effective, holistic interventions. Consequently, it is common to hear of unhelpful approaches such as “period pain is an ordinary part of being a woman,” implying it needs to be tolerated.^29,34^ Dysmenorrhoea, affecting 71% of women younger than 25 years,^2^ is a common pain that exemplifies the interplay of female-specific biological and psychosocial factors. Positive psychological framing is further impeded by fears of pelvic pathology, such as ectopic pregnancies or gynaecological cancers. A UK government survey of ∼100,000 women revealed that many often feel the need to repeatedly present to health professionals and self-advocate for adequate investigation of gynaecological issues.^9^ This fear, even when no pathology is present, is likely to make women more attentive to, and more troubled by, their “ordinary” period pains.

**Figure 1.**
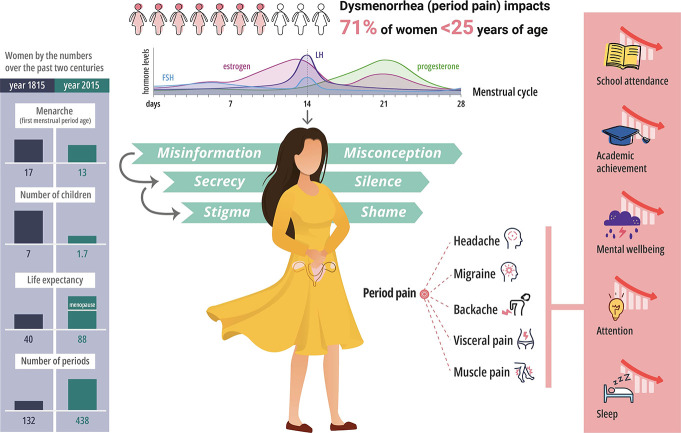
This schematic highlights the number of times that women menstruate during their lifetime and the proportion of young women who experience dysmenorrhea. The impact on academic performance, school attendance, mental wellbeing, and lifestyle is represented.

These “ordinary pains” may be commonplace in women, but they are not trivial. Period pain is a leading cause of school absences among women^2,29,34^; it has also been linked to mental health issues, sleep and attention problems, and reduced academic performance^1^—factors which reduce day-to-day quality of life and long-term outcomes in women. Adding to the burden of dysmenorrhoea are other types of pain that are commonly comorbid—including headache, migraine, and backache—and associated symptoms of menorrhagia, tiredness, nausea, and mood disturbance.^19,20^ The frequency and impact of dysmenorrhea are presented in Figure 1, highlighting an important unmet medical need. Changing societal attitude toward treating pain in women, particularly in girls and adolescents,^7,8^ in women who are older,^13^ and from ethnic minorities,^5,13^ must be addressed.

## 3. Gender differences across the human pain system

Although historically not always apparent to researchers working in the field, pain mechanisms differ between men and women, both in humans and in other animals. Researchers working with mice discovered that aspects of paw sensitivity, well established in male mice (then the standard sex for rodent pain research), were absent in female mice under specific experimental conditions.^28^ This prompted scientists to question why most pain research had previously been conducted on male subjects and to re-evaluate the notion that pain mechanisms were broadly similar between the sexes.^18^ Research has now elucidated gender differences across the human pain system from the functioning of the peripheral sensory nerves, to processing in the spinal cord and brain, to interactions between neurological and immunological cells, and to basic and higher order brain functions that account for emotional and cognitive responses to pain.^18^ These differences are mediated by complex interactions between genetics, physiological factors such as hormone changes, and how women and men are socialised to adapt to pain.^18^ Hormone changes at puberty are associated not only with the onset of female-specific pains but also widen the gender prevalence gap in other conditions, such as migraines, which only become more common in women after puberty.^31^ A further consequence of the historical dominance of pain research being conducted in men is that pain associated with the female reproductive system is less well-researched, and underlying pathologies—and nonpathological pains—are less well-understood.^10^

Given the gender heterogeneity in pain pathways, it is reasonable to consider whether women and men respond differently to analgesics. This is an area that requires further study, but there is some evidence that morphine has a greater analgesic effect in women.^17^ On average, women use more analgesics than men.^22,32^ For non-opioid analgesia, analysis of efficacy by sex is generally lacking for most common medications; however, the evidence for ibuprofen, from a meta-analysis of dental trials, suggests efficacy is broadly similar for women and men.^3^ Women may be slightly more susceptible to gastrointestinal side-effects from nonsteroidal anti-inflammatory drugs,^35^ though the differences are small and further research is required.

## 4. Effect of societal bias on women's pain experience

Apart from biological differences, there are societal differences between women and men that contribute to unconscious bias and the gender pain gap. Women are often perceived as emotional regarding pain experience, whereas men are viewed as brave and stoic.^24^ Pain in women is more frequently attributed to psychological causation, meaning women are left with the impression that their pain experience is “in their head,” and they are stigmatised as “malingerers” or “heart sink patients.”^24^ Even for pains that signal serious pathologies, such as chest pains, missed or delayed diagnoses are a greater problem in women than men.^15^ The stereotype that women are more likely to exaggerate their pain can lead observers, regardless of sex, to dismiss verbal and nonverbal expressions of pain and to underestimate women's pain.^25,36^ Women feeling dismissed by clinicians, along with stigma and lack of knowledge, are key barriers to seeking health care.^16^ Consequently, conditions requiring immediate attention may be prolonged, with treatment sought only when severity can no longer be ignored, by which point body systems may already be negatively impacted. For example, the British Heart Foundation has raised awareness about gender bias in managing myocardial infarction.^6,33^ Medical practice must improve these outcomes, but the gender pain bias is not specific to clinical practice, it operates at all levels of society.^24^

Unconscious bias is deeply ingrained in society, and bridging the gender pain gap is a complex challenge. For example, lack of awareness and communication regarding female-specific pains, such as period pain, has created structural challenges for women in schools and workplaces. Stigma associated with gynaecological conditions can lead to isolation of women during pain experiences, whereby they no longer benefit from community support or empathy, both of which can influence the efficacy of pain-relieving interventions.^14,27^ The experience of female-specific pains could be reduced by addressing exacerbating factors, eg, by improving privacy in toilets (particularly in schools), access to free sanitary products, greater flexibility within the education system or the workspace, and increased attention to gender-specific needs that could alleviate or provide better support. Trusted information is key to combating myths and misinformation. Conversations about female-specific pains may feel difficult to conduct in a public setting, so many women turn to online resources—in the United Kingdom, asking family and friends (74%) is the most common source of health information for women, but Google searches (71%) are a close runner up.^9^ Although sites such as nhs.uk offer reliable information, health information in platforms such as TikTok and Instagram lack medical oversight. Society could, and should, do better. Clinicians, researchers, regulators and content creators could all play crucial roles in improving health literacy around female pain.

## 5. Interventions in pain management in women

In medical education, the concept of the “hidden curriculum”^11^ has gained prominence, referring to the unofficial and often unintended learnings acquired in the coffee rooms and corridors of medical institutions, through mechanisms such as negative role-modelling, which usually run counter to formal and intended teachings. This is where values and behavioural norms are transferred from one medical generation to the next.^11^ Aspects of unconscious bias, including pain bias, are most likely to be propagated through the hidden curriculum. Its teachings are more powerful because they operate by stealth, through latent learnings. Hidden curricula exist not just in clinical institutions, but in all environments where learning takes place and, arguably, we can all be socialised into gender pain bias through the hidden curriculum of life. One way to counter this curriculum is to unmask it and take a compassionate approach to everyone with pain, regardless of sex, ethnicity, age, or any other aspects of diversity. Unmasking the gender pain gap and bringing it to wider public attention is the purpose of the recent “See My Pain” media pain campaign, launched by industry, in partnership with academic and charitable institutions. The campaign advocates for more empathic and validating approaches to pain in women and spotlights the problem of women's pain experiences being too often dismissed. Surveys of approximately 5000 people in the United Kingdom, run for 2 consecutive years in 2022 and 2023, indicate that 1 in 2 women report having their pain dismissed by others.^21^ These insights echo key messages in the UK Government's Women's Health Strategy for England, a 10-year plan that aims to improve the health of girls and women more generally and that draws attention to the problem of the “male as default” approach which has blighted research, health professional training, and the design of health services.^9^

Jodie Halpern, a US psychiatrist, advocates for an approach of “compassionate curiosity,” arguing that this is the key to connecting clinicians to patients and thus improving diagnosis and management.^12^ This approach encourages us to spend time with people in pain and to ask questions that help us understand their perspectives, ideas, concerns, and expectations. Compassion is a component of empathy, an attribute which the famous psychologist Carl Rodgers characterised more as an attitude than a skill: as “a way of being” with another person.^23^ Approaching women in pain with empathy and an attitude of being compassionately curious could do much to improve the current management of pain in women.

## 6. Conclusion

For biological, psychological, and sociological reasons, pain in women is not identical to pain in men. Women experience more pain than men, but society—community, schools, family and friends—is more likely to take the view that pain is a more emotional construct in women, compared with men.^24^ Female pain is taken less seriously, even for concerning pain presentations such as cardiac pain,^6,15^ and female-specific pains are complicated by stigma, hesitance to seek treatment, and a lack of accessible, quality information. Gender pain bias is deeply rooted in societal norms and stereotypes and propagated through a ‘hidden curriculum' of learning. Closing the gender pain gap will require us to unmask that hidden curriculum and engage in a co-ordinated and sustained endeavour, but that should not deter us from the attempt. Transferring learnings from empathy and compassion scholarship to the diagnosis and management of pain presentations and collaborations between governments, industry, clinicians, academics, charities, patient groups, and more will unite key stakeholders and maximise the future chance of success.

## Disclosures

W.L. is employed by the Reckitt group of companies, which owns and distributes the Nurofen brand. This paper is based on the outputs of 3 virtual meetings that formed an advisory board on aspects of pain and analgesia which go beyond pharmacology. K.V. and R.S. were participants in the advisory board, which was funded by the Reckitt group of companies. No authors were compensated for writing this Perspective.
